# A Proposed Dynamic Pressure and Temperature Primary Standard

**DOI:** 10.6028/jres.095.005

**Published:** 1990

**Authors:** Gregory J. Rosasco, Vern E. Bean, Wilbur S. Hurst

**Affiliations:** National Institute of Standards and Technology, Gaithersburg, MD 20899

**Keywords:** dynamic calibrations, dynamic sources, molecular transducer, nonlinear optical spectroscopy, pressure, primary standard, Raman spectrum, temperature, transducers

## Abstract

Diatomic gas molecules have a fundamental vibrational motion whose frequency is affected by pressure in a simple way. In addition, these molecules have well defined rotational energy levels whose populations provide a reliable measure of the thermodynamic temperature. Since information concerning the frequency of vibration and the relative populations can be determined by laser spectroscopy, the gas molecules themselves can serve as sensors of pressure and temperature. Through measurements under static conditions, the pressure and temperature dependence of the spectra of selected molecules is now understood. As the time required for the spectroscopic measurement can be reduced to nanoseconds, the diatomic gas molecule is an excellent candidate for a dynamic pressure/temperature primary standard. The temporal response in this case will be limited by the equilibration time for the molecules to respond to changes in local thermodynamic variables. Preliminary feasibility studies suggest that by using coherent anti-Stokes Raman spectroscopy we will be able to measure dynamic pressure up to 10^8^ Pa and dynamic temperature up to 1500 K with an uncertainty of 5%.

## 1. Introduction

With modern laser diagnostic techniques, it is possible to characterize the pressure (*P*) and temperature (*T*) of a gas at the molecular level. The measurement times for these techniques are such that the response to changes in *T* and *P* is limited only by the fundamental relaxation and transport processes of the molecular system. This provides the basis for a new approach to the calibration of transducers used in the measurement of dynamical *P* and *T*. The essence of dynamic calibrations is the determination of the time dependent response of the transducer, which requires, at a minimum, the application of a stimulus with known time dependence, i.e., a “standard” dynamic source.

If one were to rely on conventional sensors (whose response functions are *not a priori* known) to characterize the dynamic source, an inescapable circularity emerges from the preceding paragraph. Approaches to solution of this problem have traditionally [1] relied on some form of calculable source. In essence, this is a source some properties of which can be determined from accurate measurements, for example of quasi-static values of *P* and *T* and time rate of change of position, and whose time dependent *P* and *T* is then derived from an appropriate theoretical prescription, e.g., from hydrodynamics for sound propagation or fluid mechanics for shock waves (with appropriate equations of state for isentropic or adiabatic expansions). It must be recognized that every theory relies to some degree on idealizations and that any laboratory realization of a dynamic source is non-ideal. Thus, sources of dynamical *P* and *T* cannot be accurately known from theory alone; measurement of the “standard” source always is required.

Ideally, in the maintenance of national standards one seeks to relate the measured quantity to a constant of nature, maintained, for example, in the energy levels of an isolated atom or molecule. We are proposing this type of approach for the development of a “standard” source for dynamic *P* and *T*. The essence of our approach is to combine the very best in calculable generators, fast transducers, and high-speed digital data acquisition systems with a new, fundamental measurement approach. The latter relies on the use of laser-based diagnostic techniques, developed over the past 10 years, to determine the *P* and *T* of the dynamic system. The unique characteristics of the optical techniques are:

- *T* and *P* are derived from measurement of the optical transitions between the atomic or molecular energy levels of the constituents of the dynamic source, i.e., the atoms or molecules are the fundamental transducers of the local *P* and *T* environment
- optical measurements can be accomplished with a single laser pulse of nanosecond duration, with the consequence that the “response time of the transducer” reduces to the equilibration time for the atoms or molecules (in the interaction region) to respond to changes in the local thermodynamic variables
- optical measurements can be accomplished within harsh environments by means of transmitted or reflected laser beams and, for multiple beam techniques, spatial resolution within the source volume can be defined by the regions of overlap of these beams, e.g., mm^3^ dimensions.

Our approach relies mainly on the use of nonlinear Raman spectroscopies, since these have consistently been shown to provide useful diagnostic spectra in very short times with high spatial resolution[2]. The spectrum determined with these nonlinear Raman approaches is the simplest, best understood, and most highly characterized of any optical diagnostic technique. Comparisons of spectra observed for systems in known (static) states of *P* and *T* with the predictions of theory provide a high degree of certainty in the use of these data for *P* and *T* measurement.

The purpose of this paper is to describe the nonlinear Raman optical measurements that can provide the new primary standard for dynamical *P* and *T*. This description will include information on the *P* and *T* dependence of the spectrum and a brief consideration of the important elements of a measurement system which can be applied to a dynamic source. For the purposes of this discussion we do not consider the dynamic source in any detail; however, the information we present is considered applicable to a suitably designed shock tube source. The state-of-the-art in optical diagnostics is now at a point where accurate measurement of such a “standard” dynamic source is possible. Accuracy limits of the order of *5%* for the metrologically significant range of Pup to 10^8^ Pa and *T* up to 1500 K appear achievable.

In the following, we begin with an operational description of the use of nonlinear Raman spectroscopy for *P* and *T* measurement, drawing from the already established data base on the *T* and *P* dependence of observed spectra. We will then outline the elements of a measurement system for a dynamic source. Areas needing significant instrumental development are included in this discussion. Some questions with regard to the *P* and *T* dependence of the Raman spectrum which need further fundamental research also are highlighted. The presentation style is intended to be descriptive rather than rigorous; for completeness, more detailed information on the *T* and *P* dependence of nonlinear Raman spectra is included in the Appendix.

## 2. Nonlinear Raman Optical Diagnostics

The proposed approach to measurement of the *T* and *P* of the dynamic source is coherent anti-Stokes Raman spectroscopy (CARS)[3]. In its most simple realizations this technique uses two lasers, termed the pump and the Stokes beams, whose frequency difference is selected to be in resonance with a pure vibrational transition of a diatomic gas molecule contained in the source medium. The nonlinear interaction of the electric fields of these lasers with the molecules of the source medium generates a third, laser-like beam, termed the anti-Stokes beam, which carries the information about the molecular system, in particular about its *T* and *P*.

**Figure 1:**
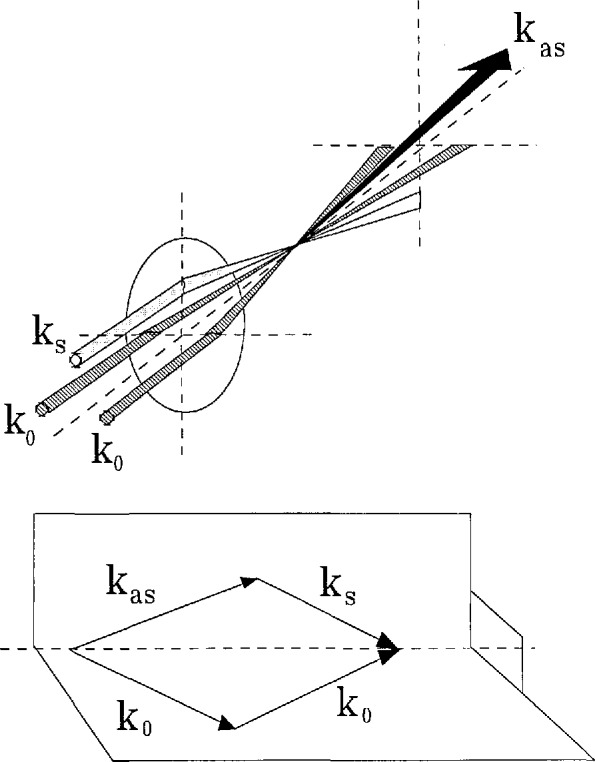
The approximate geometrical arrangement of the pump, (subscript 0), Stokes (subscript s), and generated anti-Stokes (subscript as) beams in a CARS experiment. The sample region is at the intersection of the crossing beams. The phase matching condition for (folded) BOXCARS [11] is indicated.

A useful arrangement of these beams, which provides a high degree of spatial resolution, is shown in figure 1. In this configuration, termed BOX-CARS, the interaction volume is defined by the region of overlap of the two pump beams, *k*_0_ (derived from one laser source), and the Stokes beam, *k_s_*. Sample volumes of millimeter and submillimeter dimension are remotely accessible in this arrangement.

The information about the local *T* and *P* environment of the molecules in the interaction volume is determined from the spectrum of the generated anti-Stokes beam (designated by *k*_as_ in fig. 1). The spectrum is obtained by measuring the power of the anti-Stokes beam as a function of the frequency difference between the pump and Stokes lasers. Considering for the moment a static system, a spectrum can be obtained by measuring this power as we change the frequency difference between a tunable narrowband Stokes-laser and a fixed frequency narrowband pump-laser. Since this is simply a power measurement, we can essentially eliminate the use of traditional spectroscopic instruments (e.g., prism or grating spectrometers) and retrieve an undistorted measure of the information imparted by the molecular system. The narrowband Stokes and pump laser sources can readily be made of essentially delta-function-like bandwidth for this application.

The special conditions for static systems and narrowband lasers, described in the last paragraph, have been achieved in the laboratory in order to determine the fundamental molecular response, i.e., its spectrum, under known conditions of *T* and *P*. This has been accomplished for certain ranges of these variables and for a few selected molecular systems [4]. We will illustrate the basics of spectroscopic *T* and *P* determinations by describing spectra derived from these studies.

## 3. Temperature Dependence of CARS Spectra

**Figure 2:**
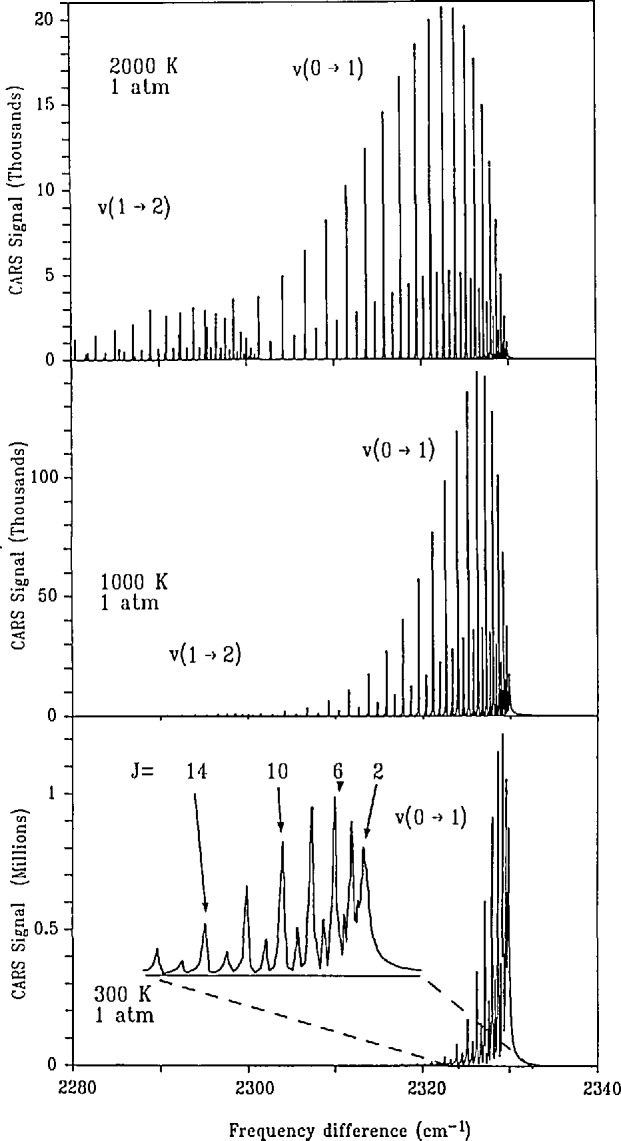
Calculated CARS spectra for the N_2_ vibrational *Q* branch as functions of *T* for fixed *P* (= 1 atm). The horizontal axis is the frequency difference between the pump and Stokes lasers. The vertical axis is a measure of the CARS power. Although the absolute units of this power are arbitrary, the relative magnitudes as a function of *T* are accurately represented. Selected transitions and bands of the complete spectrum are indicated. In the bottom panel, the spectral region from *Q*(16) thru *Q*(0) is shown on an expanded frequency scale.

**Figure 3:**
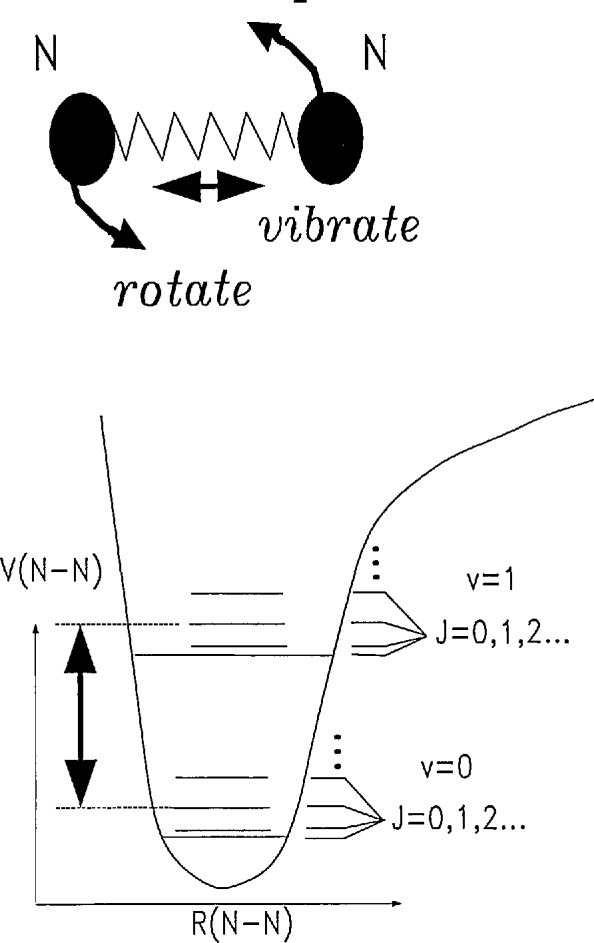
The solid curve schematically represents the potential energy of the ground electronic state versus internuclear separation. Vibrational and rotational energy levels (quantum numbers *ν* and *J*, respectively) also are indicated (not to scale). A molecular transition, *Q*(2), associated with the vibrational *Q* branch is indicated by the arrow.

In figure 2 we show CARS spectra of pure N_2_ as a function of *T* with the pressure held fixed at 1.0 atm. The horizontal axis is the frequency difference between the pump and Stokes lasers and the vertical axis is the (calculated) power in the anti-Stokes beam (in an unspecified arbitrary unit). These spectra are referred to as vibrational *Q*-branch spectra, because the optical transition involves a change only in the vibrational quantum state (quantum number *ν*) and no change in rotational state (i.e., no change in the rotational quantum number, *J*) [5]. The relevant states and modes of motion are schematically illustrated in figure 3.

Returning to figure 2, we see that there are many maxima in the power as a function of frequency difference. Each of these arises from a pure vibrational transition which originates in a different rotational state *J*. The vibrational frequency depends, to a small degree, on the rotational state because the rotation of the molecule results in a slight stretching of the bond length producing a small change in the forces binding the molecule and a concomitant change in the vibrational frequency. The vibrational frequency (we use the traditional spectroscopic unit cm^−1^, 1 cm^−1^≃30 GHz, in the figure) is approximately 2329.91 cm^−1^ for the *J*=0 rotationless state; the value decreases approximately according to 0.01738*J*(*J*+l), which places the *J*=10 transition at ≃2328.00 cm^−1^, less than a 0.1 % change. The strength (integrated area) of an individual transition is a function of the population difference between the initial (*ν* =0, *J*) and the final (*ν* = l, *J*) states of the transition. This population dependence in the relative strengths of the transitions as a function of the rotational level, *J*, is the basis for temperature determination, since for systems in thermodynamic equilibrium the state populations are functions only of the temperature. The vibrational *Q*-branch spectrum is very useful for measuring *T* because there are essentially no corrections to apply in order to relate the strength of a fully resolved transition to the population differene and therefore to the *T* [6]. As is seen in figure 2, the transitions for higher-*J* states increase in strength with increasing *T*; this simply mimics the population shifts to higher energy states associated with increasing *T*.

Strictly speaking, the temperature determined from the relative populations of the rotational levels should be called a “rotational temperature.” In like manner, a “vibrational temperature” can be determined from a measurement of the relative populations of the vibrational levels. We observe in the higher-*T* spectra in figure 2 that there are spectral maxima for transitions labeled *ν* = l→*ν* = 2. This is a vibrational *Q* branch which originates in the first excited vibrational level, *ν* = 1, and terminates in the second excited level, *ν* = 2. At sufficiently high *T* there is a significant population in the *ν* = 1 state and this transition becomes observable. This transition is totally analogous to that discussed above which initiated in the vibrational ground state, *ν* = 0. Comparison of the integrated areas between the 1*→*2 and 0→1 transitions gives a measure of the “vibrational temperature.” The rotational temperature of the vibrationally excited state also can be determined. The assumption of local thermodynamic equilibrium can thus be tested, since all levels should yield the same thermodynamic temperature in the equilibrium situation.

## 4. Pressure Dependence of CARS Spectra

First we note that the integrated area of the entire spectrum is a function of the number of molecules with which the intersecting laser beams interact. This feature often is used as a means of measuring species concentration in a diagnostic environment [2,3]. Each molecular species, e.g., N_2_, O_2_, CO, H_2_, etc., has a separate vibrational resonance because the resonant frequency is a sensitive function of the binding forces and the masses of the atoms comprising the molecule. Thus, intercomparison of the areas of these different *Q* branches can be used as a measure of the relative numbers of each molecule in the sample volume. Because it is very difficult to make accurate measurements of absolute intensity, it has been found that the absolute intensity of a transition is not a good measure of the density or pressure of a sample.

**Figure 4:**
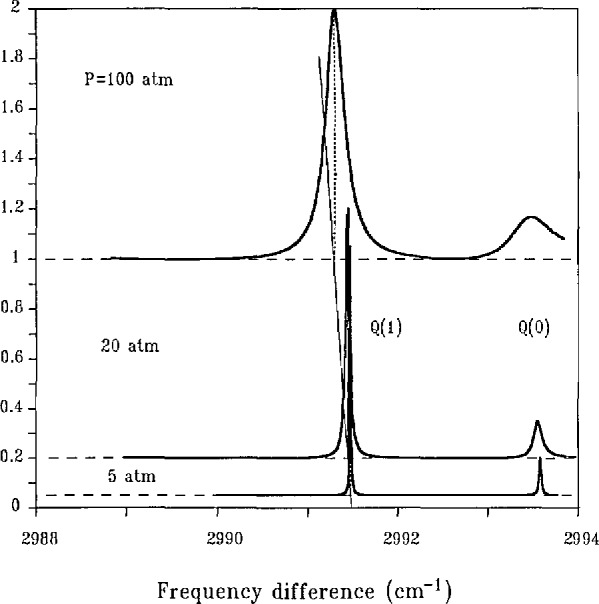
Calculated CARS spectra for the *J* = 0 and *J* = 1 transitions of the vibrational *Q* branch of pure D_2_ at *T* = 295 K. The zero of intensity for each *P* is shifted by an amount proportional to *P*. The dotted vertical lines indicate the resonance frequency of the *Q*(1) line at each *P*. The solid sloping line drawn through the vertical lines is thus an indication of the linear with *P* shift of the resonance frequency. At *P* = 0 the *Q*(0) line is at 2993.57 cm^−1^ with the higher *J* transitions at lower frequencies approximately by the amount 1.056J(*J* + 1).

Fortunately, there are good measures of the pressure of a sample which can be recovered from the *Q*-branch spectrum of some diatomics. We illustrate these by spectra of the *Q* branch of D_2_, which has been extensively studied in our laboratory. Figure 4 presents CARS spectra calculated from results of these experimental studies. The features of primary interest in this figure are the resonance frequencies and widths of the transitions. Figure 4 illustrates the important fact that these transitions change their width (broaden) and their resonance frequency (shift to lower values) with increasing pressure. In the pressure range shown, this broadening and shifting are linear with pressure. For the *Q*(1) transition of D_2_, the broadening rate is 0.0012 cm^−1^/atm (we use the conventional half width at half peak height as our measure of width) whereas the shift rate is —0.0019 cm^−1^/atm, i.e., the line shifts more rapidly than it broadens. We see also that the transitions in the D_2_ *Q*-branch spectrum remain isolated, non-overlapped, up to 100 atm. We thus identify a very appealing approach to pressure measurement in that it is tied to the measurement of the frequency positions and widths of molecular transitions.

The situation described above for the D_2_ *Q* branch should be contrasted with the observations of pressure broadening and shifting in the *Q* branch of N_2_. The broadening rates of the N_2_ transitions are typically 30–40 times larger than that of the D_2_ *Q*(1) transition. A shifting rate for a N_2_ transition is typically 10 times smaller than its broadening rate, thereby making accurate frequency determination less certain with increasing pressure. Additionally, we note that the interline spacing in the N_2_ *Q* branch is approximately l/60th that of D_2_. As a consequence, a transition such as *Q*(10) would broaden to overlap most of the other transitions shown in figure 2 at a pressure of 100 atm at room temperature. This overlap of transitions leads to important changes in the appearance of the spectrum which are discussed briefly in the Appendix. At this point it suffices to say that the N_2_ *Q* branch involves a complicated spectral distribution function which has the consequence that pressure determinations would generally be less reliable than those derived from the approach based on the *Q* branch of D_2_.

To this point we have restricted consideration to static systems and very high resolution measurements with narrowband lasers. We have displayed the results of careful measurements under known, static conditions of *T* and *P* which demonstrate that temperature can be determined by the measurement of the relative intensities of molecular transitions and that pressure can be determined from the positions and widths of molecular transitions. We turn now to discuss briefly the measurement approach required to characterize a dynamic system.

## 5. Single Shot Diagnostic Measurements

First we point out that we are interested in very rapid measurements, in the range of microseconds or less, in order to characterize the full bandwidth of transducers. Next we note, from the previous discussion, that a relatively large amount of spectral information must be acquired in order to characterize *T* and *P*. These requirements of time and spectral range (at relatively high resolution) result in the selection of a technique in which all the spectral elements are measured at once, i.e., a single shot measurement. Single shot measurements are the strong suit of nonlinear Raman diagnostics because we can employ pulsed laser sources and obtain high optical power levels which, by virtue of the inherent nonlinear response of these techniques, results in the generation of strong, readily detected, CARS signals.

The most straightforward approach to single shot measurement is to replace the scanned, narrowband Stokes laser, considered above, with a broadband Stokes laser whose frequency width is sufficient to provide the entire Raman spectrum of interest in a single shot. For example, the entire 60 cm^−1^ region in figure 2 would be measured. We note that a similar spectral bandwidth would be required for temperature measurement via the D_2_ *Q* branch because of the larger interline spacing in the D_2_ *Q* branch, cf. figure 4. The measurement of the spectrum then requires multiple detectors coupled to an instrument which disperses different frequency components in the anti-Stokes beam to the separate spatial locations of the detectors. This combination of a spectrometer and detector we refer to as a multichannel system.

## 6. Instrument Development

The basic tools for these single shot measurements do exist. The necessary laser systems have been realized in the research laboratory. Adaptation of these to specific diagnostic situations will be required. Similarly, a large variety of spectrometer/detector systems are available and this should allow for reasonably straightforward realization of the specific systems required for our application. Because of the requirements for large spectral ranges and high resolution, noted above, some multiplexing of multichannel systems will be necessary. More details on the instrumentation are discussed in the following.

### 6.1 Lasers

The basic requirements for the laser sources are well within the state-of-the-art of laser technology. The combination of a single-frequency, pulsed Nd:YAG oscillator with a series of amplifiers and a frequency doubling crystal will provide an adequate Raman pump laser. This laser also will serve as a pumping source for amplifying the required broadband dye laser for the Stokes beam. The oscillator for this Stokes laser will require special design, tailored specifically for the transitions selected for optical characterization of the dynamic source.

### 6.2 Spectrometer/Detector System

This system also will be tailored to the transitions chosen. It will be unique in that it will have a high resolution (0.001−0.05 cm^−1^) system for frequency and linewidth determination along with the more conventional low resolution (0.1−0.5 cm^−1^) channel for temperature determinations. A high resolution multichannel system has not yet been attempted in diagnostic applications. Our ability to selectively add a component such as D_2_ to serve as a pressure transducer in the working medium allows us to consider this approach. Incorporating a significant fraction of N_2_ in this medium allows for a number of independent checks on the temperature and on the state of thermodynamic equilibrium. Depending on the pressure range, we may be able to determine the temperature from the D_2_ spectrum. An approach under consideration is to simultaneously measure the *Q* branch of D_2_ for pressure determination and the pure rotation *S* branch (transitions within a vibrational state involving a change of +2 in the rotational quantum number only) of N_2_ to determine the temperature [7].

The discussion in the last few paragraphs indicates one area of the research necessary to develop a standard source of dynamic *P* and *T*. There are additional fundamental studies required to assure accurate knowledge of the *T* and *P* dependence of CARS spectra and ultimately to enable the selection of a measurement approach optimized for application to the dynamic source. We turn now to a brief discussion of these.

## 7. Fundamental Studies

The most important fundamental question to be answered in this work is the dependence of the spectra of our *specific* dynamic system on *T* and *P*. What is unique and new about the system we propose is that it is by necessity a mixed gas system, for example D_2_ contained at low concentrations in N_2_. Except for our recent studies (see ref. [4] and below), there is relatively little work on mixed gas systems. Additionally, we are interested in these systems over quite large ranges of *P* and *T*.

There are a number of motivations for the selection of a mixed gas system. First, no one molecule presents all the necessary spectral characteristics which allow an adequate characterization of both *T* and *P*. Of the many single component systems considered, D_2_ comes closest to fulfilling this requirement. D_2_ provides a good measure of *P* because it has very narrow, isolated lines. This characteristic makes it difficult to simultaneously measure the relative strengths of all the *Q*-branch transitions to obtain an accurate measure of *T*. Furthermore, it is much harder (i.e., it takes much longer) to establish thermodynamic equilibrium for D_2_ than it is for N_2_ because of the much larger gaps between the energy levels in D_2_. Large departures from thermodynamic equilibrium can degrade the performance of a dynamic source and the accuracy of the spectroscopic measurements themselves. The use of pure D_2_ has a number of other limitations; for example, a shock tube dynamic source would be very limited in dynamic range by the use of such a light gas. We note also that the cost of running a pure D_2_ system would be prohibitive, as also might the potential safety hazards associated with pure D_2_ at high pressures or high temperatures.

The use of a mixed gas system is potentially an advantage for pressure measurements. This statement is derived from our observation that mixed gas systems such as D_2_:X, with X = He, Ar, and N_2_, have a larger shift to width ratio than pure D_2_. There remain some fundamental questions in this regard, however, because we have observed line shape asymmetries in the spectrum of D_2_:Ar and H_2_:Ar [8]. We also know that these asymmetries are functions of *T*. These observations need to be fully understood in order to reliably calibrate *P*; furthermore, these studies must be extended to the D_2_:N_2_ system. An adequate spectral model and the pertinent molecular parameters must be determined and tested over the complete range of *P* and *T* of interest for the dynamic source.

## 8. Characterization of the Dynamic Source

### 8.1 Accuracy Levels in *T* and *P*

The accuracy level for dynamic pressure measurements using the proposed optical/molecular approach is estimated to be approximately 5%. This estimate is based on our ability to measure the widths and shifts of the D_2_ *Q*-branch transitions. For the mixed gas systems proposed as standards, the lines shift at least as rapidly than they broaden. Thus our accuracy goals, imply that we can locate the line center to approximately l/10–l/20th of the line width and that we can make line-width measurements at approximately the 5% level. Achieving these measurement accuracies in a single shot experiment is one of the development goals of this work.

Accuracy in temperature measurements depends on our ability to measure the relative intensities of spectral features associated with different molecular quantum states. In practice, this comes down to comparing an observed spectrum to predicted spectra such as those shown in figure 2. In a single shot experiment this comparison must include the effects of a finite-resolution, spectroscopic instrument function. The accuracy of these comparisons for single shot measurements can be of the order of 5% [3,4]. This is the target level of accuracy for temperature measurements on the dynamic source. The simultaneous and independent measurement of pressure will help eliminate systematic errors in either of these measurements.

### 8.2 Temporal Evolution

It is important to recognize that the nonlinear laser diagnostic techniques which allow us to obtain a 10 ns “snap-shot” of the local *T* and *P* environment of a molecule, can not easily be extended to provide a continuous-in-time measurement (“movie”) of the evolution of *T* and *P* in a dynamic source. These techniques do provide a primary standard which can be used to calibrate measurement approaches which yield good relative measures of *T* and *P* with rapid and continuous temporal response. Until this point, we have had no primary standard which could assure the accuracy of these measurements. The primary standard would be used to accurately pin the *P* and *T* values at representative points in the temporal evolution of the source during each calibration run. Further, the nonlinear optical techniques would be used to hold the absolute calibration of the source over long periods of use.

## 9. Conclusions

We have described a new approach to the measurement of *T* and *P* of a dynamic source. The measurement is based on the fundamental properties of molecules, specifically on the energies and populations of the vibrational and rotational levels of diatomic gas molecules. The nonlinear optical technique, coherent anti-Stokes Raman spectroscopy (CARS), has been proposed as the method for acquiring the information from the molecular system. This technique has the advantages of very rapid (nanosecond) measurement times, small sampling volumes, and a well understood and verified *T*- and *P*-dependent spectrum. The development of this measurement system to provide a reference standard for dynamic calibrations of *T* up to 1500 K and *P* up to 10^8^ Pa with a 5% accuracy appears highly feasible.

## Figures and Tables

**Figure 5:**
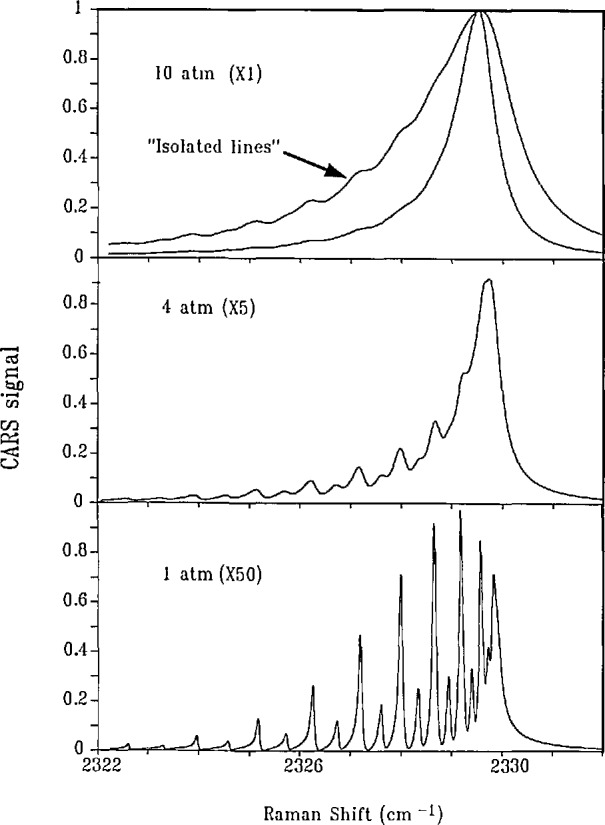
Calculated CARS spectra for the pure N_2_ *Q* branch at 295 K. The intensity at each pressure has been multiplied by the indicated factor and plotted on a unit-normalized scale. An isolated line model which considers only the diagonal elements of the relaxation matrix is included for comparison at 10 atm.

**Figure 6:**
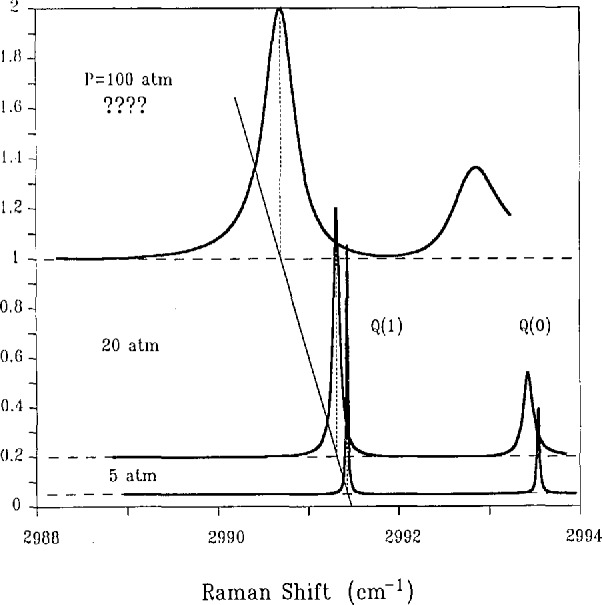
Calculated CARS spectra for the *J*=0 and *J*= 1 transitions of D_2_ contained at 10% concentration in Ar at 295 K. The spectra are based on experimental measurements which extend only up to 50 atm. The extrapolation to 100 atm is based on the lower pressure data. See caption and compare to figure 4.

## 10. Appendix

### 10.1 Raman Scattering

The Raman spectrum [5] arises from inelastic scattering in which an incident photon of frequency *ν*_0_ is “absorbed,” a scattered photon of frequency *ν*_0_±∆*ν* is “emitted,” and the molecule undergoes a transition among its internal states. The energy difference between the scattered and incident photons, h∆*ν*, is equal to that between the internal states of the scattering molecule. More concisely, Raman scattering, as we will consider it in this discussion, will refer to a two-photon transition originating in the ground electronic state, with intermediate states being far off-resonance, one-photon-allowed electronic transitions. The initial and final states of this transition are molecular vibration-rotation levels in the ground electronic state.

### 10.2 Nonlinear Optics

The fact that Raman scattering is a two-photon transition makes it essentially a nonlinear optical phenomenon. Many nonlinear Raman spectroscopy techniques simply involve impressing (on the molecules) optical fields corresponding to the two photons of the Raman transition. In the following we will briefly discuss Raman scattering in the context of nonlinear optics [3,9]. In this theory the response of a macroscopic system to an applied electromagnetic field, *E*, is written in a form such as

$$\prod \;\propto \;\chi^{\left(1\right)}E+\chi^{\left(2\right)}E:E+\chi^{\left(3\right)}E:E:E+\ldots$$ (1)

where II is the polarization density induced by the action of the fields and χ^(^*^n^*^)^ is the *n*th order susceptibility (a tensor of rank *n* +1). The relationship in eq (1) is intended to indicate that we are concerned with vector quantities and that the response of the system involves, in general, all orders of the electric fields. The electromagnetic fields and polarization densities appearing in this expression typically are assumed to be monochromatic (angular frequency ω) plane waves (wave vector *k*) with the representation

$$E=\sum_{i}\;1/2E\left(\omega_{i}\right)\exp\left(k_{i}\cdot r-\omega_{i}t\right)+c.c.$$ (2)

(c.c. denotes complex conjugate) with

$$k_{i}=\frac{n_{i}\omega_{i}}{c}$$ (3)

where *n_i_* is the index of refraction and *c* the speed of light. With this representation of the fields and polarization, the susceptibilities are written as functions of the frequencies of the associated monochromatic components of the field. Nonlinear Raman effects occur when the frequency differences and electric field directions of the applied fields can drive a Raman transition.

### 10.3 Coherent Anti-Stokes Raman Spectroscopy

In particular, the nonlinear Raman effect known as coherent anti-Stokes Raman spectroscopy (CARS) arises from the 3rd-order terms with electric field components *E_j_*(ω_0_), *E_k_*(*ω*_0_), and [*E_l_*(ω_as_)]* (the *j,k,l* are spatial indices and the “*” denotes complex conjugate) such that a polarization density at frequency *ω*_as_ with wave vector *K*_as_ is generated with the conditions

$$\omega_{\text{as}}=2\omega_{0}-\omega_{s}$$ (4a)

and

$$K_{\text{as}}=2k_{0}-k_{s}.$$ (4b)

These equations are essentially statements of conservation of energy and momentum. The latter condition indicates that a phase matching geometry must be established between the incident “pump” field (*ω*_0_) and the incident “Stokes” field (*ω*_s_) in order to generate a signal beam, the anti-Stokes component (*ω*_as_). The corresponding susceptibility has 2-photon, Raman resonances when

$$\omega_{0}-\omega_{s}=\Delta \omega_{\text{molecular}}$$ (5)

with ∆ω_molecular_ being a molecular transition angular frequency (2*π*∆*ν*=∆*ω*_molecular_).

### 10.4 Raman *Q*-Branch Resonance

For example, the fundamental (i.e., *ν* = 0→*ν* = 1) vibrational *Q* branch is composed of all transitions from the ground to first excited vibrational level without change in rotational quantum number; the molecular transition frequency in this case is

$$\Delta \omega_{\text{molecular}}=\hbar^{-1}\left[E\left(v=1,J\right)-E\left(v=0,J\right)\right].$$ (6)

This relation yields a multiple line spectrum for the *Q* branch because the rotational energy level spacing changes with the vibrational state. The transition frequencies for the vibrational *Q*-branch of a simple diatomic molecule such as N_2_, CO, or D_2_ can be expressed in terms of familiar molecular constants [5] as

$$\nu \left(J\right)≃\nu_{0}-\alpha_{e}J\left(J+1\right)+\beta_{e}J^{2}{\left(J+1\right)}^{2}$$ (7)

with ν_0_ the frequency of the pure vibrational transition between the rotationless (*J*=0) states and with the α_e_ and *β*_e_ expressing, respectively, the change in the moment of inertia and (lowest order) centrifugal distortion with vibrational state. In keeping with convention, we will express frequency in the spectroscopic unit the wavenumber, cm^−1^, which is the frequency in Hz divided by the speed of light in cm/s [*ν/c* = ω/(2*πc*)].

### 10.5 CARS Susceptibility

The CARS susceptibility resonance for the isotropic vibrational *Q* branch with laser, Stokes, and anti-Stokes electric field vectors in parallel orientations is defined by the following relations [10]

$$\begin{array}{l} \prod_{1}\left(\omega_{\text{as}}\right)=3\chi_{1111}^{\left(3\right)}\left(-\omega_{\text{as}},\omega_{0},\omega_{0},\omega_{s}\right)\\ \;{\left[E_{1}\left(\omega_{0}\right)\right]}^{2}\left[E_{1}\left(\omega_{s}\right)\right]* \end{array}$$ (8)

$$\chi_{1111}^{\left(3\right)}=\frac{iNc^{4}}{12\hbar \omega_{s}^{4}}\frac{\partial \sigma }{\partial \Omega }S\left(\omega \right)+\chi^{\text{NR}}$$ (9)

$$S\left(\omega \right)=l^{t}{\left(G\right)}^{-1}\;\Delta \rho_{0}\;l$$ (10)

$$G=G\left(\omega \right)=i\left\{\omega \;I-\omega_{J}\right\}+N\;W$$ (11)

$$\omega =\omega_{0}-\omega_{s}$$ (12)

$${\left(\omega_{J}\right)}_{JJ^{\prime }}=2\pi c\;\nu \left(J\right)\;\delta_{JJ^{\prime }}$$ (13)

$${\left(\Delta \rho_{0}\right)}_{JJ^{\prime }}=\left[\rho_{\text{eq}}\left(\nu =1,J\right)-\rho_{\text{eq}}\left(\nu =0,J\right)\right]\delta_{JJ^{\prime }}$$ (14)

$$\frac{\partial \sigma }{\partial \Omega }=\frac{\omega_{s}^{4}}{c^{4}}{\left|\sum_{r}\left\{\frac{\mu_{fr}\mu_{ri}}{\hbar \left(\omega_{ri}-\omega_{0}\right)}+\frac{\mu_{fr}\mu_{ri}}{\hbar \left(\omega_{fr}+\omega_{0}\right)}\right\}\right|}^{2}$$ (15)

with *N* the number density, *l* a column vector of 1’s (superscript “t” signifying transpose), *I* the unit matrix, (*ω_J_* and ∆*ρ*_0_ diagonal matrices of transition frequencies and (equilibrium) population differences respectively. A nonresonant contribution, χ^NR^, to the third order susceptibility (see below) is included in eq (9). Equation (10) is a short-hand matrix equation for the frequency dependence of the (Raman) resonant part of the susceptibility which involves the relaxation matrix *W*. This matrix carries all the information about the effects of collisions on the spectrum; we will discuss it later.

### 10.6 Raman Cross-Section

First we note that eq (15) is an approximate expression for the Raman scattering cross-section. It explicitly shows the two, one-photon dipole matrix elements, *μ*_mn_, connecting the initial (*i* = {*ν* = 0,*J*}) and final (*f*={*ν* = 1,*J*}) molecular levels via intermediate electronic states, *r* [the subscripts {*mn*} stand for sets such as {*ri*} or {*fr*} in eq (15)]. This form of the cross-section is used because all photons (laser, Stokes, and, anti-Stokes) satisfy the relation

$$\omega_{s}<\omega_{0}<\omega_{\text{as}}\ll \omega_{\text{mn}}$$ (16)

with

$$\omega_{mn}\equiv \hbar^{-1}\left[E\left(m\right)-E\left(n\right)\right].$$ (17)

As a consequence of these conditions, no denominator in eq (15) is near zero, i.e., we are far from any one photon resonance.

The quantity inside the squared-magnitude symbols in eq (15) is an expression for the optical polarizability matrix element for the transition {*if*}. The optical polarizability is a 2nd-rank tensor quantity which, for the systems of interest here, has two irreducible tensor components. These are a scalar, rotationally invariant part of rank zero, and a trace-less, symmetric 2nd-rank tensor part, known as the anisotropy. Reference to the “isotropic *Q* branch” indicates consideration of that part of the interaction associated with the scaler part of the polarizability matrix elements.

The squared-magnitude of the matrix element of the scaler portion of the polarizability is linearly dependent on the vibrational quantum number but essentially independent of the rotational quantum number [6]. As a result, the contribution of an individual *Q*(*J*) transition to the isotropic *Q* branch is almost exactly proportional to the population difference in eq (14).

### 10.7 Nonresonant Contribution to χ

Reference to eq (11) will show that the contribution of the Raman resonance to the susceptibility has both real and imaginary components. Far off the Raman resonance, when | ω − *ω_J_* | ≫ | *NW_J′J_* | for all {*J*′,*J*}, this contribution becomes pure real, cf. eq (9). When one includes all the possible field combinations implicit in eq (1), there are a large variety of far off-resonance two-photon transitions which add together to provide a constant-in-fre-quency real susceptibility called the nonresonant background. This is the source of χ^NR^ in eq (9).

### 10.8 CARS Intensity

The polarization density in eq (8) is a source term in Maxwell’s equations which are solved to yield the anti-Stokes field. For copropagating (along z), plane wave, linearly polarized (along *x*) pump and Stokes excitation, the approximate solution is a plane, anti-Stokes wave, with electric field parallel to *x*(*x* ≡ l)

$$E_{x}^{\text{as}}=E\left(\omega_{\text{as}},z\right)\;\exp\left(k_{\text{as}}z-\omega_{\text{as}}t\right)$$ (18)

$$\begin{array}{l} E\left(\omega_{\text{as}},z\right)=\frac{i\pi \omega_{\text{as}}}{2cn_{\text{as}}}\chi^{\text{CARS}}{\left[E\left(\omega_{0}\right)\right]}^{2}\left[E\left(\omega_{s}\right)\right]*\\ \;\frac{\sin\left(\delta kz/2\right)}{\left(\delta k/2\right)}e^{i\delta kz/2} \end{array}$$ (19)

with

$$\chi^{\text{CARS}}=12\chi_{1111}$$ (20)

and with

$$\delta k=K_{\text{as}}-k_{\text{as}}$$ (21)

expressing the phase matching condition. The wave vectors on the RHS of eq (21) are those of the CARS polarization, eq (4b), and the generated, radiative CARS field, eq (18). The intensity (power density) associated with this field is

$$I\left(\omega_{\text{as}}\right)=\frac{16\pi^{4}\omega_{\text{as}}^{2}}{c^{4}n_{0}^{2}n_{s}n_{\text{as}}}{\left|\chi^{\text{CARS}}\right|}^{2}\;I\left(\omega_{s}\right){\left(\frac{\sin\left(\delta kz/2\right)}{\delta k/2}\right)}^{2}.$$ (22)

Equations (18)–(22) involve some idealizations and approximations, such as plane wave excitation and weak field limits, which can easily be removed in the theory or to some extent achieved in the laboratory, or both, see for example reference [3], These equations do provide a basis for understanding the essential features of the signal generation process, namely, an intensity proportional to: the squared magnitude of a third order nonlinear susceptibility, χ(−ω_as_, ω_0_, ω_0_, −ω)_s_), the square of the pump intensity, the probe intensity, and a phase matching factor.

### 10.9 Spatial Resolution and Phase Matching

For systems with δ*k*≃0, e.g., low density gases, spatial resolution and signal enhancement is achieved simultaneously in the copropagating geometry, discussed above, by using focussed pump and Stokes lasers. Gaussian transverse-mode lasers yield nearly Gaussian anti-Stokes beams generated from spatial volumes of diameter less than the pump focal diameter and with axial distances of the order of 6z_0_, with z_0_ the familiar confocal parameter of the focussed pump beam [3]. For a 5-mm diameter (*d*_0_) pump focussed by a 20-cm (*f*) focussing lens, the approximate dimensions of the sample volume are found from the equations for the focussed beam diameter, 2*w*_0_,

$$2w_{0}≃\frac{4\lambda_{0}}{\pi }\frac{f}{d_{0}}=28\times 10^{-4}\;\text{cm}$$ (23)

and for the confocal parameter

$$z_{0}=\frac{\pi w_{0}^{2}}{\lambda_{0}}=0.108\;\text{cm}.$$ (24)

Spatial resolution can be enhanced by crossing the pump and Stokes beams, for example as shown in figure 1. The requirements of phase matching for signal enhancement, c.f. eqs (21) and (22), result in the folded BOXCARS [11] arrangement shown in the figure. This arrangement will yield a spatial resolution nearer to the dimensions 2*w*_0_ and 2*z*_0_. Since the folded BOXCARS geometry yields good spatial separation of the anti-Stokes beam, this usually is the spectroscopically preferred arrangement. However, many other configurations are possible [2].

### 10.10 CARS Spectrum

The spectrum results from the frequency dependence of the squared magnitude of χ. From eqs (9) and (10), we can write the general form

$${\left|\chi \right|}^{2}={\left(\chi^{\text{NR}}\right)}^{2}+2{\chi^{\prime }}_{R}\chi^{\text{NR}}+{\chi \prime_{R}}^{2}+{\chi {\prime \prime }_{R}}^{2}$$ (25)

with the subscript R referring to the (Raman) resonant part of the susceptibility and the prime denoting real and the double prime denoting imaginary part, respectively. If we limit consideration to a single, presumed isolated (our specific meaning for this term will be clarified below) transition, at *ω_J_*, the resonant part of the susceptibility is

$$\chi_{R}\;\propto \frac{\left(\omega -\omega_{J}\right)+i\;\Gamma_{J}}{{\left(\omega -\omega_{J}\right)}^{2}+{\Gamma_{J}}^{2}}$$ (26)

with Γ*_J_* the linewidth of the pressure broadened line. The sum of the last two terms in eq (25) yields a Lorentzian lineshape, i.e.,

$$I\left(\omega \right)\;\propto \frac{1}{{\left(\omega -\omega_{J}\right)}^{2}+{\Gamma_{J}}^{2}}.$$ (27)

The presence of a nonzero nonresonant background leads to asymmetric lineshapes via the second term in eq (25). The first term yields a uniform nonzero signal level which can be dominant if the concentration of the resonant species is low.

### 10.11 Collisional Line Interference

A transition can be considered isolated if the condition expressed by the relation

$$\sum_{J^{\prime }\neq J}\frac{W_{J^{\prime }J}}{\left(\omega_{J^{\prime }}-\omega_{J}\right)}\ll 1$$ (28)

is satisfied. The quantity *W_J′J_* is an off-diagonal element of the relaxation matrix introduced in eq (11). It is a measure of the rate of collisionally-induced exchange between transitions (see below). Equation (28) simply states that if the rate of exchange between transitions is much smaller than the frequency separation of the transitions, then they can be considered isolated. Conversely, if collisions are mixing lines at a rate commensurate with their separations, we will see line interferences. These are described by the full relaxation matrix equation. The corrections for multiple line spectra which arise when we cannot ignore the nondiagonality of the relaxation matrix in eq (11) are discussed in the following.

### 10.12 The Relaxation Matrix

#### 10.12.1 Pressure Shift and Broadening

In general, the diagonal elements of the relaxation matrix describe the familiar pressure broadening and shifting of spectral lines [12]. Thus, the resonant frequency and linewidth in eq (26) are

ωJ=ΩJ+δJP (29)

[with the convention that the quantity Ω*_J_* is the zero pressure value derived from accurately known molecular constants, see for example eq (7)] and

$$\Gamma_{J}=\gamma_{J}\;P.$$ (30)

Equations (29) and (30) implicitly define pressure shift and pressure broadening coefficients, δ and *γ* respectively.

The change and spread of the observed resonance frequency are associated with intermolecular collisions. Encounters with other molecules alter the binding forces and therefore the resonance frequencies of the optically active molecule. In the pressure regimes of our interest, such encounters are limited to binary collision events such that the duration of the encounter is much shorter than the time between collisions. In simplest terms, the shifting coefficient expresses the average rate of collision-induced phase shift of the oscillator. Similarly, the broadening coefficient expresses the rate at which the oscillator is dephased by the collisions.

These effects are expressed as proportional to pressure because the probability per unit time that a collision produces a phase shift in the observed oscillation frequency can be written as the product *N·ν*_av_·*σ* of the number density, *N* (molecule/cm^3^), the average velocity, *ν*_av_ (cm/s), and a cross section, *σ* (cm^2^), appropriate to shifting or broadening for the collision. Such an expression is analogous to that for the collisional momentum transfer which defines the pressure in the gas.

We now turn to consideration of the off-diagonal elements of the relaxation matrix.

#### 10.12.2 Line Mixing and State-to-State Rates

In the familiar “impact limit” of collision physics (aspects of which were enumerated in the preceding section, see also ref. [12]), the elements of *W* are proportional to appropriately averaged products of two scattering (“*S*-matrix”) operators. For molecules such as N_2_ and CO, it has been shown that there is very weak dependence of the scattering matrix elements on the vibrational states. This reveals itself in very weak shifting and a line broadening which is independent of vibrational (e.g., 1→2 vs 0→1) or overtone (e.g., 0*→*2 vs 0→1) branch. The absence of strong vibrational state dependencies in the *S*-matrix elements yields a relaxation matrix for the isotropic *Q* branch which can be expressed entirely in terms of state-to-state rates for rotational energy transfer. The off-diagonal element, *W_J′J_* for these conditions, is simply the negative of the inelastic rate for a transition from *J* to *J′*. In terms of the discussion in the last section, we can see that the occurrence of an inelastic collision terminates the oscillation, i.e., equivalently totally dephases the oscillator. Very little shifting would be expected under these conditions. Thus, the diagonal term, the line broadening coefficient, reduces to the total inelastic rate out of a state *J*.

Because of the nonzero off-diagonal components, the spectrum resulting from the relaxation matrix equation will show the phenomenon of collisional narrowing of the *Q* branch with increasing pressure. The important criterion for the narrowing is a rapid rate of rotational energy transfer relative to the frequency difference between the *Q*(*J*) transitions, i.e., the relation in eq (28) is not satisfied. Systems such as N_2_ and CO show significant narrowing at 1 atm and room temperature, because they have relatively small rotational energy gaps with concomitantly large rates of rotational energy transfer, and small interline spacings within the *Q* branch. In contrast, the spectra of D_2_ displayed in figure 4 can be considered isolated, independent lines. We illustrate the effects of interference in the following.

### 10.13 *Q* -Branch Spectra of N_2_

Pressure dependent spectra of pure N_2_ at room *T* are shown in figure 5. For comparison, an isolated line model (*W_J′J_* = 0 for *J*′≠*J*) for the spectrum at 10 atm is also included in the figure. Clearly, neglect of the collisional interference terms, *W_J′J_*, would lead to large errors in pressure determinations. The accuracy of theoretically predicted spectra, such as those shown in figure 5, has been tested against spectra measured on samples in the pressure range up to 100 atm and for temperatures from 295 up to 2400 K (the upper limit in *T* depends on *P* because of the pressure limits of high-*T* furnaces and cells), see reference [4].

### 10.14 Energy Gap Laws for Rotational Energy Transfer

The fundamental basis for calculation of the spectra shown in figure 5 is an energy-gap rate law [13] for the individual state-to-state rates for rotational energy transfer. As discussed above, knowledge of the state-to-state rates allows specification of the entire relaxation matrix and therefore computation of the spectrum [from eqs (9)–(11) and (22)]. The most widely used form of the rate law for specification of the relaxation matrix is the modified exponential gap (MEG) law [14] which parameterizes the upward *(J→J′* with *J*<*J′*) state-to-state rate according to

$$\begin{array}{l} W_{J^{\prime }J}=\alpha_{0}\;f\left(T\right)\cdot {\left(\frac{1+A\;E\left(J^{\prime }\right)/kT\delta^{\prime }}{1+A\;E\left(J^{\prime }\right)/kT}\right)}^{2}\\ \;\cdot {\left(\frac{1+A\;E\left(J\right)/kT\delta }{1+A\;E\left(J\right)/kT}\right)}^{2}\cdot \exp\left(\frac{-\beta \left\{E\left(J^{\prime }\right)-E\left(J\right)\right\}}{kT}\right) \end{array}$$ (31)

with

$$f\left(T\right)={\left(\frac{T_{0}}{T}\right)}^{1/2}\frac{1-\exp\left(-m\right)}{1-\exp\left(-mT/T_{0}\right)}.$$ (32)

The downward rate is found from the principle of detailed balance, i.e.,

$$\rho_{\text{eq}}\left(J\right)W_{J^{\prime }J}=\rho_{\text{eq}}\left(J^{\prime }\right)W_{J^{\prime }J}.$$ (33)

The quantity *A* is calculated from the Lennard-Jones intermolecular potential parameters for each molecular collision system. The parameters α_0_, δ′, δ, *β*, and *m* are determined from comparisons to accurately measured line broadening coefficients, *γ_J_*, (functions of *J* and *T*) and to experimentally determined pressure dependent spectra.

There remain some significant research issues in the prediction of N_2_ isotropic *Q*-branch spectra, such as: some refinement of the rate law model seems important at high temperature [15] and the appropriate spectral and rate law model for situations where N_2_ is found in a foreign gas host must be experimentally examined.

### 10.15 Q-Branch Spectra of the Hydrogens

The state-of-the-art for prediction of the spectrum of molecular systems such as D_2_(or H_2_):X [X=D_2_(or H_2_), He, Ar, N_2_] is not as fully developed as for N_2_:N_2_. This situation arises because there is a large vibrational state dependence in the *S*-matrix elements. Such a vibrational state dependence leads to the addition of a significant “elastic vibrational dephasing,” discussed earlier, contribution to the linewidth and line shift in these systems. The elastic vibrational dephasing component, although understood formally from theory, is not easily calculated and does not have a simple predictive basis similar to that just presented for the rotationally inelastic component.

The significance of elastic dephasing for the hydrogens is judged relative to their rotationally inelastic contributions. For the hydrogenic systems, the presence of elastic vibrational dephasing is evidenced by pressure shifting coefficients, δ_J_ [cf. eq (29)], which are of the order of and often a few times larger than the line broadening coefficients, γ_j_ [cf. eq (30)]. Because of the large rotational energy gaps in these systems the inelastic portion of the total line broadening is relatively weak (especially for the foreign gas collision partners). Small inelastic rates and large vibrational dephasing both lead to a decrease in the line interference resulting from the solution of eqs (10) and (11). Finally, there is large rotation-vibration interaction in the hydrogens, e.g., α_e_ in eq (7) is 1.08 for D_2_, more than 60 times larger than the α_e_ of N_2_. As a result of all these factors, the hydrogens will display essentially isolated-line type spectra with shift and width measurements yielding good measures of the pressure. The spectra displayed in figure 4 illustrate the pressure sensitivity of the pure gas system, those in figure 6 indicate the potential sensitivity for the D_2_:Ar system. The spectra given in this figure are based on actual measurements which to this point have been extended only to 50 atm. Realizing the potential of this system requires more study as we will discuss in the following.

The complete *J*, *P*, and *T* dependence of the spectra of D_2_(H_2_):X is yet to be determined. Some preliminary data on pure H_2_ up to 1000 K and 50 atm has been obtained. An empirical fitting law based on the MEG law for the rotationally inelastic contribution and a simple power law in *T* for scaling the (assumed *J* independent) vibrational dephasing contribution has been applied with some success to these data. More work is needed for all the systems of interest, in order to have a predictive basis.

In particular there remain some important scientific questions with respect to the foreign gas systems. Recent work has revealed anomalous concentration dependent lineshapes for the D_2_(and H_2_):Ar system [8]. These anomalies are associated with a large (collision) speed dependence in the cross-sections for line shifting and a small propensity of D_2_:Ar collisions to result in speed changes. As a result, the lines are inhomogeneously broadened and rather asymmetric at very low concentrations of the active molecule in the Ar host. More study of the concentration, pressure, and temperature dependence of the D_2_:Ar lineshape is required.

### 10.16 Doppler Broadening

The line broadening, shifting, and interferences discussed above are appropriate to pressure and temperature regimes in which the rate of velocity changing collisions, Nν_av_σ_D_, with σ_D_ the “diffusion cross-section,” is larger than the Doppler width, *≈|k_0_−k*_s_|*ν*_av_, of the Raman transition. Doppler broadening arises from the free streaming of the molecules, and, at low pressures, it can be used for measurement of translational temperature. For the hydrogens, in particular, this source of line broadening is significant at pressures up to 10 atm at high temperatures, e.g., 1000 K. Research on the D_2_:X systems (X = D_2_, H_2_, He, CH_4_, Ar), under conditions where both velocity changing and normal pressure broadening collisions, as discussed above, contribute to the line shape is in progress. A reliable theoretical description appears possible, especially for relatively light collision partners, using a simple diffusion-like model solution to kinetic equations for line formation. However, more work, in particular for the heavy collision partners and high temperatures, remains to be done for these systems.

### 10.17 Summary

In the above, we have briefly summarized essential aspects of the theory of nonlinear Raman spectroscopy as seen from the viewpoint of one who wishes to apply the technique to temperature and pressure measurements. This has involved some discussion of elements of the theory of spectral line formation for the relevant Raman transitions. Although the level of detail and rigor of this discussion has been limited, the intention is simply to indicate that we have a good understanding of the basic physics which underlies our measurement approach. A great deal of work has gone into building this understanding and developing a reliable predictive basis for temperature and pressure measurements. It is now possible to transfer the static pressure and temperature scale to the molecular level to serve as a primary standard for dynamic *P* and *T*.
